# Level of self-care practices and associated factors among hypertensive patients in Addis Ababa, Ethiopia

**DOI:** 10.1186/s12872-023-03062-9

**Published:** 2023-01-25

**Authors:** Addisu Tadesse Sahile, Hayat Abdulkadir Nurhussien

**Affiliations:** 1 Department of Public Health, Unity University, Addis Ababa, Ethiopia; 2 Department of Out Patient, Bole Bulbula Health Center, Addis Ababa, Ethiopia

**Keywords:** Self-care practice, Predictors, Hypertension, Addis Ababa

## Abstract

**Objectives:**

The study assessed the level of self-care practice and its predictors among hypertensive patients in the health centers of Bole Sub-city, Addis Ababa, Ethiopia.

**Methods:**

A multi-Center-based cross-sectional study that employed 370 hypertensive participants at the conveniently selected Health Centers in Bole Sub-City; from August 01–30, 2020. The researchers selected the participants based on a simple random sampling method after applying for a pre-tested interviewer-administered questionnaire and secured for informed consent. All the statistical analyses were SPSS 22.0 software based. The authors used binary logistics regression to identify the presence and strength of association; with its respective 95%CI and p-value less than five percent as a significant level.

**Results:**

The overall level of good self-care practice among hypertensive patients was 53.0% (95% CI: 47.2–58.8%) whereas 61.4%, 63.8%, 92.7%, 82.7%, and 18% of the study participants were adherent to medication, good weight management, non-smokers, alcohol abstainers and physical activity consecutively. Being illiterate had 2.347 and 2.084 times higher odds of having had good self-care practice compared to secondary school and a diploma or above consecutively. Being a merchant, civil, and retired were associated with good self-care practice than being unemployed.

**Conclusion and recommendation:**

The study reported a lower level of self-care practice in the study settings. Educational level and occupation were factors identified for self-care practice. The authors recommended policymakers, healthcare workers, and researchers work on the identified factors of self-care practice of hypertensive participants in the study settings.

**Supplementary Information:**

The online version contains supplementary material available at 10.1186/s12872-023-03062-9.

## Introduction

Hypertension; “the silent killer” is the top public health problem of both developed and the undeveloped world [1–6] affecting 1.13 billion adults globally [7]; and was expected to rise up to 1.56 billion people by the year 2025 [8]. It has an annual mortality rate of 9.4million deaths per year [9] incurring 10% of the global health expenditure [10].

Based on the recommendation by the World Health Organization (WHO), hypertensive patients should monitor their blood pressure (BP) after trained about the measurement procedure [11]. Self-care practice has a pivotal role in the prevention and control of raised blood pressure [12, 13]. Commonly; the self-care practice includes maintenance of healthy body weight (Body Mass Index; BMI) 18.5 to 24.9, medication adherence and waist circumference, moderate-intensity physical exercise; 30–60 min for 4–7 days a week, dietary approach(low salt diet; moderate alcohol consumption; and cessation of smoking) and stress management [14, 15].

With the premise that the self-care practice by the hypertensive patient should encompasses maintenance of body weight, adherence to medication, waist circumference, physical exercise, dietary management and stress management, there was a global effort to reach at by the year 2025. These included reduction of the raised blood pressure by 25%, alcohol use and insufficient physical exercise by 10%, non-communicable disease by 25%, and population salt-intake and tobacco use by 30%. With all this efforts, different studies reported a lower level of self-care practice [16–18].

An optimal self-care practice controls BP, and other cardiovascular disease related morbidities and mortalities [19]. Though there were efforts to combat non-communicable diseases, the level of self-care practice among hypertensive patients was not a well-investigated area. Hence, this study assessed the level of self-care practice and its associated factors among hypertensive patients in selected health centers in Addis Ababa, Ethiopia, and would fill the existing gap.

## Methods

### Participants and study design

**Fig. 1:**
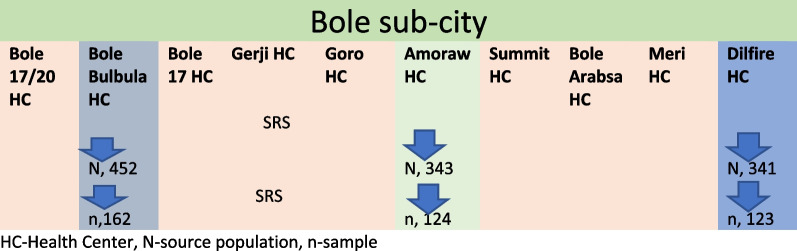
Sampling technique for the study

A multi-center-based cross-sectional study that received ethical approval from Santé Medical College, research review and ethics committee and Addis Ababa Regional research Review Committee and was conducted at the selected health centers in Addis Ababa, the capital of Ethiopia that has 10 sub-cities with 96 health centers. The researcher selected Bole sub-city, within Addis Ababa city, with a total of10 health centers providing healthcare service to about 415,572 population based on the report from the sub city. Proportionally, the sub city has 52% of female population. The researchers selected Bole sub-city conveniently, with the premise that healthcare service delivery system and procedure was similar across all the sub-cities within Addis Ababa. In Bole sub-city, there are ten health centers, five of which were included in the study based on simple random sampling method. The selected health centers were Bole Bulbula health center, Delfire health center and Amoraw Health Center (Fig. 1).

The researchers carried out the study from August 01 to 30, 2022; ones secured a written informed consent from all the study participants following the approval from institutional review board (IRB) of Santé Medical College and research center of Addis Ababa city health bureau. The source population were all Hypertensive patients in Bole Sub-city, whereas the study population included 370 hypertensive patients, on follow-up and available during the data collection period at the selected Health centers fulfilling the inclusion criteria. Hypertensive patients should be on follow-up at least for six months in the selected health center, not critically by the time of data collection to respond to the questionnaire, and have no mental illness diagnosed clinically to be included in the study. Patient selection followed the principles of simple random sampling technique, ones obtained for their list from the respective health centers medical record. The sample size was determined based on population proportion estimation formula, n = z^2^p (1 − p)/d^2^, with the assumptions of, 95% confidence interval (CI), 5% margin of error, and a self-care practice by hypertensive patient of 59.4% [20]. Where p-proportion of self-care practice, n-sample size and d-margin of error. The final sample size was 409, including the 10% non-response rate. Data collected with a pre-tested interviewer-administered questionnaire where a pre-test conducted on 5% of cases at Bole 17 Health Center two weeks before actual the data collection period. The questionnaire was first developed by the researchers after a rigorous review of literature [21–25] then given to senior researchers, their inputs incorporated finally.

Outcome variable measurement: Self-care practice; the participants were regarded as having had good self-care practice if they scored above the median and poor if less than the median score of the constructs making up the self-care [26].

In this particular study, self-care practice was measured by Hypertension self-care Activity level Scale effects (H-SCALE) that have six main domains; namely: medication adherence (3 items): Low-salt diet (12 items): scores of 6 out of 7 days-adherents. Physical activity (2 items): responses range from 0 to 14. Participants who scored 8 or better were considered adherent. Smoking (2 items): responses range from 0 to 14, and respondents who reported 0 days were considered a non-smoker, and all others were smokers. Weight management (10 items): responses range from 10 to 50. Participants who agreed or strongly agreed with all 10 items (score ≥ 40) were considered to have a good weight management practices. Alcohol (3 items): responses range from 0 to 21. Participants who did not take any alcohol in the last 7 days or who did not drink at all were abstainers.

### Statistical analysis

Data entry and cleaning were through Epi-Info version7, and then analyzed by statistical package for social sciences (SPSS) version 22.0 software after exported. Descriptive Statistics summarized the finding. Once the outcome variable dichotomized as poor and good self-care practice, factor identification was through binary logistics regression. After checked for model fitness, through testing for Omnibus test with a *p*-value of 0.136, R^2^ of 0.106, and Hosmer Lemeshow goodness of fit test- with a *p*-value of 0.775. Additionally, tested for multi collinearity tested with a Variance inflation factor for all the variables all showing a lower value than 0.05. Then after, selection of candidate variables done through bivariate logistics regression at a *p*-value of 0.20 in line with its respective 95% confidence interval and *p*-values. To control the effect of the confounding variable, a multivariable logistics regression was done, with its respective 95%CI and p-value less than 5% used as a level of significance.

## Results

### Socio-demographic characteristics

**Fig. 2:**
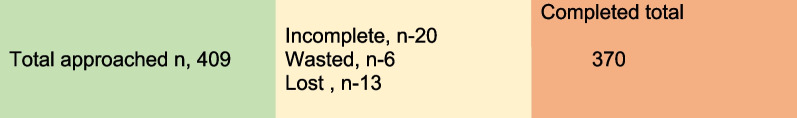
Response rate of the study participants

Totally 370 hypertensive patients approached from the Bole sub-city, and gave a response rate of 90%. The authors excluded all missed and incomplete data (Fig. 2). The mean age of the study participants was 63.39 years with a standard deviation of ± 13.569 years. More than half (55.7%) and 57% of the study participants were females and married consecutively. Most (63.8%) of the study participants had a monthly income of higher than 1000 birr (Table 1).

**Table 1:** Socio-demographic characteristics respondents in Health Centers of Bole sub-city, Addis Ababa, Ethiopia, November 2020 [n = 370]

Variables	Category	Frequency	Percentage
Age in years	< 40	24	6.5
	40–60	129	34.9
	60–80	189	51.1
	> 81	28	7.6
Gender	Male	164	44.3
	Female	206	55.7
Education	Illiterate	127	34.3
	Primary	99	26.8
	Secondary	63	17.0
	Diploma and above	81	21.9
Marital status	Married	211	57
	Divorced	77	20.8
	Widowed	82	22.2
Occupation	Housewife	96	25.9
	Merchant	50	13.5
	Civil servant	64	17.3
	Retired	146	39.5
	Unemployed	14	3.8
Income in Birr	< 500	116	31.4
	500–1000	18	4.9
	> 1000	236	63.8

### Health care related profile of hypertensive patients

Most (60%) of the study participants had no family history of hypertension. More than one-third (36.5%) of the study participants had the hypertension with a duration of less than two years. In most (64.1%) of the study participants, type of hypertension was primary with the majority (84%) of the participants did not have a Sphygmomanometer at home (Table 2).

**Table 2:** Health profile of Hypertensive Patients in Health Centers of Bole sub-city, Addis Ababa, Ethiopia, November 2020 (n = 370)

Variables	Category	Frequency	Percentage
Family history of HTN	Yes	148	40
	No	222	60
Comorbidity	Yes	138	37.3
	No	232	62.7
Duration since diagnosis	< 2 years	135	36.5
	2–4 years	102	27.6
	> 4 years	133	35.9
Hypertension type	Primary	237	64.1
	Secondary	133	35.9
Had a home sphygmomanometer	Yes	57	15.4
	No	313	84.6

### Self-care practice of hypertensive patients

Almost all (96.2%) of the study participants took blood pressure pills, and 237 (64.1%) of the participants took their medication at a constant time every day. Most (70%) of the study participants did not engage in a specific physical exercise (Additional file 1: SI). However, most (61.4%), and 63.8% of the study participants adhered to medication and good weight management practices consecutively. The majority (92.7%) and 82.7% of the study participants were non-smokers and alcohol abstainers consecutively (Table 3).

**Table 3:** Self-care practice of hypertensive patients in health centers of bole sub-city, Addis Ababa, Ethiopia, November 2020 (n = 370)

Self-care practice domains	Frequency	Percentage
Medication adherence		
Adherent	227	61.4
Non-adherent	143	38.6
Low salt diet		
Adherent	55	14.9
Non-adherent	315	85.1
Physical activity		
Adherent	67	18.1
Non-adherent	303	81.9
Smoking		
Non-smoker	343	92.7
Smoker	27	7.3
Weight management		
Good weight management practice	236	63.8
Poor weight management practice	134	36.2
Alcohol		
Abstainers	306	82.7
Not abstainers	64	17.3

### Self-care practice and its associated factors among hypertensive patients

Overall, the level of self-care practice among hypertensive patients was 53.0% (95% CI: 47.2–58.8%). Educational status, age, gender, marital status, monthly income, duration since diagnosis and occupation of the study participants were candidate variables identified in the bivariate logistics regression analysis. In the multivariable logistics regression analysis, only educational status and occupation of the study participants were statistically associated with self-care practice.

The odds of having had a good self-care practice was 2.347 and 2.084 times higher among illiterate participants compared to participants with secondary schooling (AOR: 2.347, 95% CI: 1.223–4.614, *p* < 0.05), and participants with a diploma and above (AOR: 2.084, 95% CI:1.073–4.047, *p* < 0.05) respectively.

The odds of having had a good self-care practice was higher by 56% among Merchants (AOR: 0.44, 95% CI: 0.005–0.350, *P* < 0.05), higher by 20% among Civil servants (AOR: 0.80, 95% CI: 0.10–0.627, *P* < 0.05) and higher by 5% among retired (AOR: 0.95, 95% CI: 0.12–0.751, *P* < 0.05) compared against unemployed participants (Table 4).

**Table 4:** Factors affecting the self-care practice of hypertensive patients in Health Centers of Bole sub-city, Addis Ababa, Ethiopia, November 2020 (n = 370)

Variables	Category	Good practice N (%)	Poor practice N (%)	COR (95% CI)	AOR (95% CI)
Age	< 40	14 (3.8%)	10 (2.7%)	1	1
	40–60	65 (17.6%)	64 (17.3%)	1.378 (0.571–3.329)	1.589 (0.621–4.064)
	61–80	99 (26.8%)	90 ((24.3%)	1.273 (0.538–3.008)	1.396 (0.477–4.086)
	> 81	18 (4.9%)	10 (2.6%)	0.778(0.254–2.386)	1.074 (0.285–4.049)
Gender	Male	90 (24.3%)	74(20.0%)	1	1
	Female	106 (28.6%)	100 (27.1)	1.147 (0.760–1.731)	1.122 (0.677–1.858)
Educational Status	Illiterate	78 (21.1%)	49 (13.2%)	1	1
	Primary	54 (14.6%)	45 (12.2%)	1.327 (0.778–2.261)	1.247 (0.711– 2.184)
	Secondary	27 (7.3%)	36 (9.7%)	2.122 (1.149–3.921)*	2.375 (1.223–4.614)*
	Diploma & above	37(10.0%)	44 (11.9%)	1.893 (1.076–3.329)*	2.084 (1.073–4.047)*
Marital Status	Married	106(28.1%)	105(27.8%)	1	1
	Divorced	41 (11.1%)	36 (9.7%)	0.886 (0.526–1.495)	0.991 (0.565–1.736)
	Widowed	49 (13.2%)	33 (8.9%)	0.680 (0.405–1.141)	0.649 (0.365–1.155)
Occupation	Housewife	50(13.5%)	46(12.4%)	0.368 (0.108–1.255)	0.467 (0.118– 1.839)
	Merchant	33(8.9%)	17(4.6%)	0.206 (0.56–0.755)*	0.44 (0.005–0.350) *
	Civil Servant	31(8.4%)	33(8.9%)	0.426 (0.121–1.500)	0.80 (0.10 –0.627) *
	Retired	78(21.1%)	68(18.4%)	0.349 (0.105–1.163)	0.95(0.12–0.751) *
	Unemployed	4(1.1%)	10(2.7%)	1	1
Income group	< 500	61(16.5%)	55(14.9%)	1	1
	500–1000	9(2.4%)	9(2.4%)	1.109 (0.411–2.994)	5.592 (0.863–36.231)
	> 1000	126(34.1%)	110(29.7%)	0.968 (0.620–1.511)	4.279(0.857–21.360)
Family history of HTN	No	120 (32.4%)	102 (27.6%)	0.897 (0.592–1.361)	0.750 (0.478–1.182)
	Yes	76 (20.5%)	72 (19.5%)	1	1
Duration of diagnosis	< 2 years	71 (19.2%)	63 (17.0%)	1	1
	2–4 years	57 (15.4%)	45 (12.2%)	0.890 (0.530–1.493)	0.853 (0.491–1.481)
	> 4 years	68 (18.4%)	66 (17.8%)	1.094 (0.677–1.767)	1.134 (0.680–1.893)

*P < 0.05; **P < 0.001-statistically significant association

## Discussion

In this study, the overall level of good self-care practice by hypertensive participants was 53.0%. Thus this was consistent with the findings of 51.5% in Addis Ababa, Central Ethiopia [27], 49% in Dessie town, North Central Ethiopia [21].

However, the findings from the current study reported a higher level of self-care practice than the findings of 27.3% in southern Ethiopia [18], 37.1% in central India [26], 20.3% in Tigray Region, Northern Ethiopia [28]. This variation might be due to differences in sample size, time of investigation and characteristics across the studies.

The level of self-care practice by hypertensive participants in this study was much higher than the findings from south India that reported a self-care practice of 14% [29]. The variation might be due to the differences in sample and time of the investigation between the studies.

A study from southwest of Ethiopia reported a level of self-care practice by hypertensive patient of 46.9%. Thus, which is consistent with the current finding [30]. Moreover, a study from Nepal reported a comparable proportion (52.2%) of hypertensive patients having a consistent level of self-care practice [31].

In the contrary, a hospital based-study from Addis Ababa reported a lower level of self-care practice by hypertensive patients [32]. This variation might be due to variations in sample size across the studies.

A study from Gondar, Northwestern Ethiopia, reported that about sixty percent of hypertensive participants had good self-care practice; which was higher than the findings of the current study [33]. This variation might be associated with differences in samples between the studies.

In this study medication adherence was reported among 61.4% of the study participants, thus which was lower than the other findings 83.7% in Saudi Arabia [34] and 85% in Western Nepal [35].The exhibited variation might be due to differences in time of the investigation and difference in type and level of service.

In this study low salt adherence was observed among 14.9% of the study participants, which was consistent with the finding from 18.5% in Nigeria[36] whereas much lower than the reports of 79.3% from Saudi Arabia [34] and 94.6% in Ethiopia [37]. This difference might be due to differences in the level of awareness among the population.

This study reported less than one fifth (18%) of the study participants had adhered to physical activity, which was lower than the finding of 57.3% in Saudi Arabia [34], and higher than the study in Nigeria where the level of adherence was 9.3% [36]. The study from Saudi Arabia revealed that good weight management practice was observed among 59.9% [34] which was higher than the current study.

In this study, participants with a lower/no-educational level had good self-care practice. This was in line with the other different studies [22, 28]. This study also reported that occupation to be statistically associated with self-care practice, and other study also reported the same [28].

Limitation of the study: This was a cross sectional study, establishing the temporal association was difficult on cause-effect relationship. The authors suggested a cohort study to identify factors having a direct risk on the self-care practice of hypertensive patients.

## Conclusion

This study reported a lower level of self-care practice by hypertensive patients. The educational level and occupation were factors identified having had statistically significant association with the self-care practice. Having a lower level of education, being a merchant or retired or civil servant was statistically associated with having had of lower self-care practice. The authors suggested policymakers, researchers, healthcare professionals to work on the identified problems in aggregation with every effort to prevent hypertension-related complications or promote the health of people with chronic medical conditions.

## Supplementary Information

**Additional file 1**. SI Hypertensive Patients Self-care activities in Health Centers of Addis Ababa, Ethiopia, November 2020 (n = 370).

## Data Availability

The generated finding of this study was from data collected and analyzed based on the stated methods and materials hence all data were already available in the manuscript.

## Abbreviations

AOR: Adjusted odds ratio
DBP: Diastolic blood pressure
HTN: Hypertension
WHO: World Health Organization

## References

[1] Bel K, Twiggs J, Olin B. Hypertension: The Silent Killer: Updated JNC-8 Guideline Recommendations, Associate Clinical Professor of Pharmacy Practice. Drug Information and Learning Resource Center, Alabama Pharmacy Association. 2015.

[2] Collin J, Hughes D (2011). The silent killer in media stories: Representations of hypertension as health risk factor in French-language Canadian newspapers. Health Risk Soc.

[3] Moore J (2005). Hypertension: catching the silent killer. Nurse Pract.

[4] Kalehoff JP, Oparil S (2020). The story of the silent killer. Curr Hypertens Rep.

[5] Talha J, Priyanka M, Akanksha A (2011). Hypertension and herbal plants. Int Res J Pharm.

[6] Scullin MK, Le DT, Shelton JT (2017). Healthy heart, healthy brain: Hypertension affects cognitive functioning in older age. Transl Issues Psychol Sci.

[7] Zhou B, Bentham J, Di Cesare M, Bixby H, Danaei G, Cowan MJ (2017). Worldwide trends in blood pressure from 1975 to 2015: a pooled analysis of 1479 population-based measurement studies with 19· 1 million participants. Lancet.

[8] Kearney PM, Whelton M, Reynolds K, Muntner P, Whelton PK, He J (2005). Global burden of hypertension: analysis of worldwide data. Lancet.

[9] Lim SS, Vos T, Flaxman AD, Danaei G, Shibuya K, Adair-Rohani H (2012). A comparative risk assessment of burden of disease and injury attributable to 67 risk factors and risk factor clusters in 21 regions, 1990–2010: a systematic analysis for the Global Burden of Disease Study 2010. Lancet.

[10] Gaziano TA, Bitton A, Anand S, Weinstein MC (2009). The global cost of nonoptimal blood pressure. J Hypertens.

[11] Organization WH. A global brief on hypertension: silent killer, global public health crisis: World Health Day 2013. World Health Organization, 2013.

[12] Chobanian AV, Bakris GL, Black HR, Cushman WC, Green LA, Izzo JL (2003). The seventh report of the joint national committee on prevention, detection, evaluation, and treatment of high blood pressure: the JNC 7 report. JAMA.

[13] McManus RJ, Mant J, Bray EP, Holder R, Jones MI, Greenfield S (2010). Telemonitoring and self-management in the control of hypertension (TASMINH2): a randomised controlled trial. Lancet.

[14] Mancia G, Fagard R, Narkiewicz K, Redon J, Zanchetti A, Böhm M (2014). 2013 ESH/ESC practice guidelines for the management of arterial hypertension: ESH-ESC The Task Force for the management of arterial hypertension of the European Society of Hypertension (ESH) and of the European Society of Cardiology (ESC). Blood Press.

[15] Lamb SA, Al Hamarneh YN, Houle SK, Leung AA, Tsuyuki RT (2018). Hypertension Canada’s 2017 guidelines for diagnosis, risk assessment, prevention and treatment of hypertension in adults for pharmacists: an update. Can Pharm J/Rev des Pharmaciens du Canada.

[16] Tibebu A, Mengistu D, Negesa L (2017). Adherence to recommended lifestyle modifications and factors associated for hypertensive patients attending chronic follow-up units of selected public hospitals in Addis Ababa, Ethiopia. Patient Preference Adherence.

[17] Iloh GUP, Amadi AN, Okafor GOC, Ikwudinma AO, Odu FU, Godswill-Uko EU. Adherence to lifestyle modifications among adult hypertensive Nigerians with essential hypertension in a primary care clinic of a tertiary hospital in resource-poor environment of Eastern Nigeria. J Adv Med Med Res. 2014:3478–90.

[18] Buda ES, Hanfore LK, Fite RO, Buda AS (2017). Lifestyle modification practice and associated factors among diagnosed hypertensive patients in selected hospitals, South Ethiopia. Clin Hypertens.

[19] Han H-R, Lee H, Commodore-Mensah Y, Kim M (2014). Development and validation of the hypertension self-care profile: a practical tool to measure hypertension self-care. J Cardiovasc Nurs.

[20] Worku Kassahun C, Asasahegn A, Hagos D, Ashenafi E, Tamene F, Addis G (2020). Knowledge on hypertension and self-care practice among adult hypertensive patients at University of Gondar Comprehensive Specialized Hospital, Ethiopia, 2019. Int J Hypertens.

[21] Ademe S, Aga F, Gela D (2019). Hypertension self-care practice and associated factors among patients in public health facilities of Dessie town, Ethiopia. BMC Health Serv Res.

[22] Dasgupta A, Sembiah S, Paul B, Ghosh A, Biswas B, Mallick N (2017). Assessment of self-care practices among hypertensive patients: a clinic based study in rural area of Singur, West Bengal. Int J Community Med Public Health.

[23] Adler AJ, Laar A, Prieto-Merino D, Der RM, Mangortey D, Dirks R (2019). Can a nurse-led community-based model of hypertension care improve hypertension control in Ghana? Results from the ComHIP cohort study. BMJ Open.

[24] Nwankwo GE. Reducing Blood Pressure among African American Adults Using Educational Interventions: Brandman University; 2019.

[25] Rozani M (2019). Self-care and related factors in hypertensive patients: a literature review. Dinamika Kesehatan: Jurnal Kebidanan dan Keperawatan.

[26] Bilal M, Haseeb A, Lashkerwala SS, Zahid I, Siddiq K, Saad M (2016). Knowledge, awareness and self-care practices of hypertension among cardiac hypertensive patients. Global J Health Sci.

[27] Ahmed SM. Assessment of Knowledge, Self-Care Practice and Associated Factors towards Hypertension among Hypertensive Patients in Public in Hospit Addis Ababa City Adiministration: Addis Ababa University; 2016.

[28] Gebremichael GB, Berhe KK, Beyene BG, Gebrekidan KB (2019). Self-care practices and associated factors among adult hypertensive patients in Ayder Comprehensive Specialized Hospital, Tigray, Ethiopia, 2018. BMC Res Notes.

[29] Jonaid M. Sadang^1 3^, Sittie Inshirah P. Macaronsing^2^, Narima M Alawi^2^, Normala M Taib^2^, Hamdoni K Pangandaman^1^, Nornihayah A Arnorol^2^, Norhata L Noor^2^, Paulo Carl G Mejia^3 4^, Naima D Mala^1^, Ashley A Bangcola^1^. 2020:1865–79.

[30] Melaku T, Bayisa B, Fekeremaryam H, Feyissa A, Gutasa A (2022). Self-care practice among adult hypertensive patients at ambulatory clinic of tertiary teaching Hospital in Ethiopia: a cross-sectional study. J Pharm Policy Pract.

[31] Shrestha J, Marasine NR, Lamichhane R, Marasini NR, Sankhi S (2021). Attitude and self-care practice on hypertension among antihypertensive medication users in a tertiary care hospital Nepal. SAGE Open Med.

[32] Bacha D, Abera H. Knowledge, attitude and self-care practice towards control of hypertension among hypertensive patients on follow-up at St. Paul’s hospital, Addis Ababa. Ethiopian journal of health sciences. 2019;29(4). 10.4314/ejhs.v29i4.2 PMC6689705 31447514

[33] Worku Kassahun C, Asasahegn A, Hagos D, Ashenafi E, Tamene F, Addis G, et al. Knowledge on hypertension and self-care practice among adult hypertensive patients at University of Gondar Comprehensive Specialized Hospital, Ethiopia, 2019. Int J Hypertens. 2020;2020. 10.1155/2020/5649165 PMC7191404 32373351

[34] Bakhsh LA, Adas AA, Murad MA, Nourah RM, Hanbazazah SA, Aljahdali AA, et al. Awareness and knowledge on hypertension and its self-care practices among hypertensive patients in Saudi Arabia. Ann Int Med Dent Res. 2017;2(5).

[35] Karmacharya R, Paudel K (2017). Awareness on hypertension and its self-management practices among hypertensive patients in Pokhara, western Nepal. Janapriya J Interdiscip Stud.

[36] Boima V, Ademola AD, Odusola AO, Agyekum F, Nwafor CE, Cole H, et al. Factors associated with medication nonadherence among hypertensives in Ghana and Nigeria. Int J Hypertens. 2015;2015. 10.1155/2015/205716 PMC4610060 26509081

[37] Tesema S, Disasa B, Kebamo S, Kadi E (2016). Knowledge, attitude and practice regarding lifestyle modification of hypertensive patients at Jimma University specialized hospital, Ethiopia. Primary Health Care: Open Access.

