# Supplementary material for: Level of self-care practices and associated factors among hypertensive patients in Addis Ababa, Ethiopia
Source: BMC Cardiovasc Disord. 2023 Jan 25;23:48. doi: 10.1186/s12872-023-03062-9 (PMC9875385; doi:10.1186/s12872-023-03062-9)
Supplement: Supplementary file 1 — Additional file 1. SI Hypertensive Patients Self-care activities in Health Centers of Addis Ababa, Ethiopia, November 2020 (n = 370). [file 12872_2023_3062_MOESM1_ESM.docx]

**SI Hypertensive Patients Self-care activities in Health Centers of Addis Ababa, Ethiopia, November 2020 (n = 370)**

| **Variables** | **Response** | **Number** | **%** |
| --- | --- | --- | --- |
| How many of the past 7 days did you take your blood pressure pills? | 0-3 days | 5 | 1.3 |
|  | 4-6 days | 9 | 2.4 |
|  | 7 days | 356 | 96.2 |
| How many of the past 7 days did you take your blood pressure pills at the same time every day? | 0-3 days | 77 | 20.7 |
|  | 4-6 days | 56 | 15.1 |
|  | **7** days | 237 | 64.1 |
| How many of the past 7 days did you take the recommended number of your blood pressure pills? | 0-3 days | 65 | 17.5 |
|  | 4-6 days | 64 | 17.3 |
|  | 7 days | 241 | 65.1 |
| How many of the past 7 days did you follow a healthy eating plan? | 0-3 days | 263 | 76.4 |
|  | 4-6 days | 87 | 23.5 |
|  | 7 days | 20 | 5.4 |
| How many of the 7 days did you eat >= 5 servings of fruits and vegetables? | 0-2 days | 256 | 69.1 |
|  | 3-4 days | 111 | 30.0 |
|  | 5-7 days | 3 | 0.9 |
| How many of the past 7 days did you eat store-bought or packaged bakery goods? | 0 days | 349 | 94.3 |
|  | 1-4 days | 21 | 5.6 |
|  | 5-7 days | 0 | 0 |
| How many of the past 7 days did you salt your food at the table? | 0-2 days | 309 | 83.5 |
|  | 3 -4 days | 11 | 3 |
|  | 5 – 7 days | 29 | 7.9 |
| How many of the past 7 days did you add salt to your food when you are cooking? | 0-3 days | 79 | 21.4 |
|  | 4-6 days | 55 | 13.8 |
|  | 7 days | 236 | 63.8 |
| How many of the past 7 days did you avoid eating fatty foods? | 0-3 days | 70 | 18.8 |
|  | 4-6 days | 32 | 8.15 |
|  | 7 days | 268 | 72.4 |
| How many of the past 7 days did you do at least 30 minutes total of physical activity? | 0 days | 198 | 53.5 |
|  | 1-4 days | 133 | 35.9 |
|  | 5-7 days | 39 | 10.5 |
| How many of the past 7 days did you do a specific exercise activity (such as swimming, walking, or biking) other than what you do around the house or as part of your work? | 0 days | 259 | 70.0 |
|  | 1-4 days | 106 | 28.7 |
|  | 5-7 days | 5 | 1.4 |
| How many of the past 7 days did you smoke a cigarette or cigar, even just one puff? | 0 days | 343 | 92.7 |
|  | 1-4 days | 14 | 6.6 |
|  | 5-7 days | 13 | 3.5 |
| To lose weight or maintain my weight for the past 30 days I am careful about what I eat | Strongly Disagree | 18 | 4.9 |
|  | Disagree | 64 | 17.3 |
|  | Neutral | 22 | 5.9 |
|  | Agree | 197 | 53.2 |
|  | Strongly Agree | 69 | 18.6 |
| To lose weight or maintain my weight for the past 30 days I exercise to lose or maintain my weight | Strongly Disagree | 27 | 7.3 |
|  | Disagree | 254 | 68.6 |
|  | Neutral | 5 | 1.4 |
|  | Agree | 75 | 20.3 |
|  | Strongly Agree | 9 | 2.4 |
| To lose weight or maintain my weight for the past 30 days I have cut out drinking sugary sodas and sweet tea | Strongly Disagree | 6 | 1.6 |
|  | Disagree | 31 | 8.4 |
|  | Neutral | 2 | 0.5 |
|  | Agree | 266 | 71.9 |
|  | Strongly Agree | 64 | 17.3 |
| To lose weight or maintain my weight for the past 30 days I eat smaller portions or eat fewer portions. | Strongly Disagree | 20 | 5.4 |
|  | Disagree | 108 | 29.2 |
|  | Neutral | 12 | 3.2 |
|  | Agree | 179 | 48.4 |
|  | Strongly Agree | 51 | 13.8 |
| To lose weight or maintain my weight for the past 30 days I have cut out or limit some foods that I like but that is not good for me. | Strongly Disagree | 23 | 6.2 |
|  | Disagree | 113 | 30.5 |
|  | Neutral | 12 | 3.2 |
|  | Agree | 179 | 48.4 |
|  | Strongly Agree | 43 | 11.6 |
| To lose weight or maintain my weight for the past 30 days I eat at restaurants or fast food places less often | Strongly Disagree | 23 | 6.2 |
|  | Disagree | 85 | 23.0 |
|  | Neutral | 0 | 0 |
|  | Agree | 190 | 51.4 |
|  | Strongly Agree | 72 | 19.5 |
| On average, how many days per week do you drink alcohol? | 0 days | 306 | 82.7 |
|  | 1-4 days | 56 | 15.2 |
|  | 5-7 days | 8 | 2.2 |
| On a typical day that you drink alcohol, how many drinks do you have? | A drink of alcohol | 49 | 13.2 |
|  | Two drinks of alcohol | 8 | 2.2 |
|  | Above two drinks of alcohol | 3 | 0.8 |
| What is the largest number of drinks that you have had on any given day within the last month? | A drink of alcohol | 13 | 3.5 |
|  | Two drinks of alcohol | 31 | 8.4 |
|  | Above two drinks of alcohol | 16 | 4.3 |
